# Nerve Coaptation in 2023: Adjuncts to Nerve Repair Beyond Suture

**DOI:** 10.1016/j.jhsg.2024.01.027

**Published:** 2024-04-11

**Authors:** Alexis L. Clifford, Christopher S. Klifto, Neill Y. Li

**Affiliations:** ∗ University of Florida College of Medicine, Gainesville, FL; † Department of Orthopaedic Surgery, Duke University, Durham, NC

**Keywords:** Nerve coaptation, Nerve repair, Nerve wraps

## Abstract

Effective nerve coaptation entails tensionless repair of healthy fascicles with intact fascicular architecture and a well-vascularized environment, supportive of the regenerative cellular behaviors of neurons, immune cells, and Schwann cells. Suture coaptations have historically been used to ensure that these criteria are met for end-to-end repair, nerve transfers, and allograft or autograft reconstructions; however, unfortunately, overall restoration of function remains poor. As optimal coaptation is required for return of sensorimotor function, adjunct biomaterials are increasingly being enlisted attempting to optimize these suture-based coaptations. The purpose of this review was to discuss the biological, preclinical, and clinical data for the use of fibrin glue and nerve wraps made of type 1 collagen, porcine small intestine submucosa, chitosan, and human amniotic membrane. This study provides available data on each material’s ability to optimize the regenerative potential of nerve repair as well as available outcomes data. Although each biomaterial discussed has benefits to nerve regeneration, at large, data remain heterogeneous, and continued investigation is required to fully understand the specific mechanisms involved and the long-term potential clinical impacts each can provide for improvement of sensorimotor outcomes.

Nerve coaptation techniques are foundational to nerve surgery and an area ripe for innovation. The way coaptation is conducted is critical in dictating the potential of axons to regenerate, elongate, and ultimately reach their target sensory endings and motor end plates. As nerve surgery has progressed, suture coaptations have primarily been used to ensure end-to-end nerve repairs, nerve transfers, or allograft and autograft reconstructions are provided the best opportunity to host a path toward restoration of lost sensorimotor function. However, despite skilled microsurgical techniques to deliver epineural and, when needed, fascicular repairs, recovery of nerve function remains poor. This has prompted investigations toward strategies to enhance the regenerative potential at the site of coaptation to provide patients with improved prognoses.

Optimal nerve coaptation consists of a tensionless repair of healthy fascicles from the proximal nerve around healthy soft tissues, offering an environment conducive to healing.^1^^,^^2^ Unfortunately, ensuring a tension-free repair within a regenerative microenvironment is not always possible. Under such conditions, tension reduces perfusion to the nerve, and a less hospitable environment reduces the regenerative cellular behaviors of neurons, immune cells, and Schwann cells, which are critical for axonal growth across the coaptation. The loss of a regenerative sequence further increases the risk of neuroma formation and perineurial scarring. As such, techniques and devices, which can reduce the likelihood of these complications, would be invaluable.

The purpose of this review was to highlight the current literature on proposed strategies that may assist in delivering an optimal environment for peripheral nerve regeneration during nerve coaptation. This review contains both preclinical and clinical studies on the use of fibrin glue and commercial nerve wraps and conduits made of type 1 collagen, small intestine submucosa (SIS), chitosan, and human amniotic membrane (hAM), used about the sites of coaptations to optimize and advance suture coaptation and reduce the risk of poor regenerative outcomes for patients.

## Fibrin Glue

Fibrin glue is the most used coaptation adjunct currently on the market. This biomaterial is a derivative of concentrated fibrinogen and thrombin, traditionally used as a tissue adhesive since the 1940s.^3^ This compound is both biocompatible and biodegradable with a half-life of about 4.6 days and complete degradation by 4 weeks in humans.^4^^,^^5^ Mechanistically, this sealant catalyzes the final steps in the coagulation cascade, producing an adhesive capsule capable of maintaining tissue approximation.^3^ The original rationale for its use in peripheral nerve surgery was to reduce the excess iatrogenic trauma associated with suture-based epineural repair. This, in turn, could reduce potential fibrosis around the area of coaptation and enhance the regenerative potential of the injury site.

### Preclinical and clinical studies

The commonly cited benefits for use of fibrin glue include ease and efficiency of use; however, data for the overall benefit in peripheral nerve repair remain heterogeneous. Much of the currently available data were collected from animal or cadaveric models, with studies commonly utilizing the following three different coaptation groups: fibrin glue alone, suture alone, and suture(s) augmented with fibrin glue. These studies compare electrophysiologic, biomechanical, functional, and histologic outcome variables between repair techniques.

Fibrin glue is not currently utilized in isolation for nerve coaptation. The main outcome variable that limits this is dehiscence. From a recent meta-analysis, there were zero reported episodes of dehiscence in repairs with sutures or a combination of sutures + fibrin glue across 20 studies, whereas six of 16 studies reported dehiscence in repairs using fibrin glue alone.^3^ Second, postoperative muscle mass was found to be reduced when fibrin glue was used in isolation, suggesting reduced functional recovery.^3^ These data together suggest against the utilization of fibrin glue without adjunctive microsutures.

When considering the use of fibrin glue as an adjunct, the strength of the repair is a critical metric of success. Unfortunately, mechanical load to failure studies on suture-based nerve repair as well as on the addition of fibrin glue are heterogeneous.^6^ A recent cadaveric study demonstrated significant increases in maximum force when both suture and glue were utilized.^7^ However, other biomechanical data demonstrated that suture alone did not differ significantly from suture with fibrin glue.^3^^,^^7^ This heterogeneity is complicated by a variety of differences in the tests and procedures studied. Some have suggested that the time of testing may play a role. Sutures have been shown to yield stronger repairs through day 14, when fibrin glue may become equivalent in strength as the repair ages.^8^ Others suggest that differences among the types of soft tissue trauma induced play a role, as the fibrinous reaction forms the bond between epineurium and fibrin glue, potentially resulting in increased strength.^9^ Finally, variety in duration and simulated tension may be relevant in these differences as retraction and fibrosis of nerve stumps complicate subsequent repair.^9^

Another key metric of success is the ability to produce a regenerative environment. Unfortunately, substantial heterogeneity also exists between studies on histologic outcomes, making synthesis of these data difficult. Some believe that fibrin glue reduces inflammation and scar tissue formation secondary to iatrogenic injury with microsutures and thus permits a more robust regeneration.^10^ A 2022 meta-analysis of 11 studies demonstrated no significant difference in amplitude, conduction velocity, or latency between suture repairs and fibrin glue with or without sutures.^3^ A more recent study, however, has demonstrated that concurrent use of fibrin glue and sutures produces superior axonal regeneration, analyzed through the number and quality of axons present compared with sutures alone.^11^

Functional outcomes may also provide insight into clinical implications for the use of fibrin glue. Current data do not uniformly demonstrate significant improvements in these indices with addition of fibrin glue. Systematic review found no differences in functional index or muscle strength between suture repairs and fibrin glue repairs with or without sutures.^3^ Other recent data comparing fibrin glue with microsuture also found no significant difference at 1-year post-op in both motor and sensory functions.^10^

Studies have uniformly found that the use of fibrin glue results in a substantial decrease in operative time. Data have demonstrated a 3–4 times shorter operation time with utilization of fibrin glue in isolation when compared with suture repairs.^3^^,^^10^^,^^12^ This added efficiency, through reduction in the number of microsutures required, is beneficial for both the patient and the surgeon. This suggests that although this material may add additional cost per procedure, the decrease in procedure time may, in turn, generate savings for the hospital and the payer. Future studies geared toward analyzing the cost of use of this material would be valuable to elucidate the economic impact of this adjunct.

Although this material is commonly utilized by surgeons as an adjunct in peripheral nerve coaptation, its use remains off-label, largely secondary to a lack of sufficient controlled clinical trial data for approval of this use case.^3^ Although this lack of clinical data makes analysis of the potential added benefit from this adjunct difficult, currently, data suggest that addition of fibrin glue maintains superior or noninferior outcomes to sutures alone in all outcome categories. Until further data are generated, surgeons should maintain the use of fibrin glue in conjunction with sutures. However, standardization of clinical evaluative tests is required to elucidate the true potential value added as well as the incidence of complications to better guide surgeon adoption.

## Commercial Nerve Wraps

Many biomaterials have been studied attempting to optimize nerve coaptations with critical consideration of structure design, cellular reactivity, and extracellular matrix (ECM) components. Over the continuum in the use of nerve wraps, initial indications for its use have been to reduce the risk of perineural scarring around the coaptation and potentially reduce the risk of axonal escape and neuroma formation.^13^^,^^14^ Increasingly, with advances in biomaterials, considerations for their use have also pushed toward augmenting the coaptation microenvironment to enhance nerve regeneration.^4^

There are currently six biomaterials that have been commercially available as Food and Drug Administration (FDA)–approved nerve wraps. Table 1 demonstrates currently available commercial nerve conduits with the biomaterial utilized and the year of FDA approval. Given changes in clinical practice, this review will analyze in depth the following four biomaterials: type 1 collagen, porcine SIS, chitosan, and hAM, which is FDA-exempt.

**Table 1:** FDA Approved Coaptation Devices∗

Device Name	Manufacturer	Owner/Distributor	Biomaterial	FDA Approval
NeuroTube	Synovis Micro	Baxter International	PGA	1999
NerBridge	Synovis Micro	Baxter International	PGA + Collagen	2016
SaluBridge	Salumedica	The Georgia Tech Research Corporation	PVA	2000
SaluTunnel	Salumedica	The Georgia Tech Research Corporation	PVA	2005
Neurolac	Polyganics	Regenity	D,L-PLCL	2011
NeuroMatrix	Collagen Matrix	Stryker	Collagen type 1	2001
NeuroMend	Collagen Matrix	Stryker	Collagen type 1	2006
NeuroFlex	Collagen Matrix	Stryker	Collagen type 1	2014
NeuraGen	Integra LifeSciences	Integra LifeSciences	Collagen type 1	2001
NeuraGen 3D	Integra LifeSciences	Integra LifeSciences	Collagen type 1 + Glycosaminoglycan	2014
AxoGuard	AxoGen Corporation	AxoGen Corporation	Porcine SIS	2016
Reaxon Plus	Medovent GmbH	KeriMedical	Chitosan	2015
Neuroshield	Checkpoint Surgical	Checkpoint Surgical	Chitosan	2019

D, L-PCLC, poly(DL-lactide-ε-caprolactone); PGA, polyglycolic acid; PVA, polyvinyl alcohol; SIS, small intestine submucosa.

∗ Table depicts the FDA-approved devices for nerve coaptation, their manufacturer, owner/distributer, and the biomaterial used.

## Type 1 Collagen

### Biologic basis

Type 1 collagen was introduced as a nerve conduit material in the early 2000s. Collagen is classically expressed in the ECM of the peripheral nervous system and is an attractive material for use in coaptation as it is porous, biocompatible, and absorbable between 4 and 8 months.^4^^,^^15^ Its use in peripheral nerve is based upon its potential in assisting Schwann cell migration and providing mechanical support for axonal outgrowth.^16^^,^^17^

The clinical use of type I collagen for nerve wraps is supported by data that suggest its ability to isolate nerve tissue aids in the healing process and minimize the likelihood of neuroma formation. Literature has routinely claimed that these collagen nerve wraps improve nerve gliding and decrease perineural scarring by creating a secluded environment ripe for remyelination and axonal healing.^18^^,^^19^ The porous outer layer has been proposed to mechanically resist compression from surrounding tissues, prevent scar ingrowth, and minimize the potential for nerve entrapment,^20^ whereas the semipermeable inner membrane allows for diffusion of smaller nutrient molecules and retention of growth factors.

### Preclinical and clinical studies

Despite the proposed biologic basis, limited data supporting the biologic rationale for collagen’s use as a coaptation adjunct currently exist. Studies have demonstrated heterogeneous results regarding collagen nerve wrap’s impact on nerve gliding, myelination, and axonal regeneration. Data have indicated higher peak pull-out forces, indicating worse gliding as well as no improvement in axonal density.^18^ Debate also remains regarding remyelination with some studies, indicating increased density of Schwann cells, suggesting improved myelination, and others finding no differences.^18^^,^^21^^,^^22^ Regarding perineural scar tissue formation, although collagen wraps have been demonstrated to reduce this process, these results have not translated to superior motor recovery in the animal model.^21^ Staining for α-SMA has also detected an increased presence of myofibroblasts in peripheral nerve tissues after the use of collagen wraps, indicating a high inflammatory response to injury, which may entrap the regenerated nerve fibers and lead to pain for patients.^23^

A randomized controlled trial compared collagen conduits with direct sutures for coaptation.^22^ This study demonstrated no significant differences in sensory function, discomfort, or motor scores by 24 months after surgery when utilizing collagen conduits rather than direct suture. Additionally, amplitude, latency, and conduction velocity did not differ significantly between these groups.^22^ At large, although available clinical data demonstrate that these wraps may offer convenient off-the-shelf options for nerve coaptation, further studies are required to understand their clinical efficacy and true role in this field.^24^

## Small Intestine Submucosa

### Biologic basis

Porcine SIS is another biomaterial being utilized as ECM scaffolding for tissue repair and nerve regeneration. It maintains the features of native tissue and provides several unique properties that position it as an excellent bioscaffold.^23^^,^^25^ Small intestine submucosa has a 3-dimensional structure composed of around 40% collagen with aminoglycans and glycoproteins, and a degradation profile of between 3 and 4 months.^4^ It is proposed that fibronectin plays an adhesive role for SIS, and cytokines produce a natural environment of attachment and migration, thus increasing biocompatibility and promoting restoration of function.^25^ Another proposed mechanism of action for SIS in peripheral nerve generation is through interaction with GDNF, making it capable of enhancing recovery of neuromuscular function through increased motor axon regeneration.^26^ At large, SIS has been demonstrated to induce a favorable remodeling profile and tissue response, suggestive of its ability to align axonal outgrowth and guide nerve regeneration.^27^

### Preclinical and clinical studies

Animal models have demonstrated the feasibility and safety of this material to be utilized clinically as a nerve wrap or protective barrier. Studies have included histologic assessments, evaluation of motor conduction velocity, and number of adhesions; however, outcomes data are mixed.^28^ Some data demonstrate that SIS-wrapped nerves appear both well myelinated and vascularized as well as not statistically different than sham control in regards to velocity of conduction and impression of adhesions through 6 months post-repair.^28^ Some studies analyzing maximum load of the coaptation with use of a SIS nerve conduit have also demonstrated increased load to failure compared with suture alone.^29^^,^^30^ Contrarily, other animal studies have found no observable differences in meaningful clinical outcomes such as axonal regeneration, reinnervation, or functional recovery with adjunctive use of these wraps with epineural repair, demonstrating the remaining heterogeneity of these data.^31^

A recent study comparing SIS and collagen conduits supports the use of SIS.^23^ This study demonstrates a dominant macrophage type 2 response and lymphocytes within the SIS conduit, indicating increased regenerative response following implementation. This SIS conduit also demonstrated deeper implant vascularization and fibroblast ingrowth than collagen conduits.^23^ The cumulative available preclinical data regarding SIS material being utilized for peripheral nerve regeneration is favorable; however, further clinical trials are required to understand its long-term potential clinical efficacy.

## Chitosan

### Biologic basis

Chitosan is a naturally occurring polysaccharide found in insects, crustaceans, and fungi produced by deacetylation and modification of chitin. Chitosan is widely used for a diverse set of biomedical applications because of its distinguished biocompatibility, complete biodegradation between 74 and 77 weeks, antimicrobial properties, and interaction with the ECM and growth factors.^4^^,^^32^ This scaffold material is used in tissue engineering because of the inherent modifiability of this material and its ability to support cell growth and differentiation. Chitosan has also proven to be capable of improving survival, proliferation, and activity of Schwann cells, supporting axonal outgrowth, reducing fibroblast infiltration and scarring, and producing a neuroprotective effect secondary to downstream metabolites. In vitro data have supported this, showing that Schwann cells cultured on chitosan films are viable, and dissociated sensory neurons produced neurite outgrowth, thus demonstrating the metabolic activity and proliferative nature of this material.^33^

Chitosan resorption products are additionally considered neuroprotective, encouraging the remodeling of peripheral nerves.^34^ Particularly, the resorption product, chitosan oligosaccharides (COS), is proposed to play a critical role in its ability to regenerate peripheral nerves by promoting cell proliferation and preventing apoptosis.^34^ The mechanism inherent to COS is the stimulation of Schwann cell proliferation, promotion of nerve regeneration, and construction of favorable microenvironments at the injury site.^32^ Recent data have added to this proposed mechanism demonstrating that COS upregulates a group of proteins involved in exosome packaging and transport, thus promoting axon extension and regeneration.^32^ Other data point to the positive charge of chitosan as valuable in the reduction of postoperative infections through disruption of the bacterial cell wall.^35^ As these biologic data are promising, research has sought to optimize this material by altering the degree of acetylation, finding 5% as an optimal degree for peripheral nerve repair.^36^

### Preclinical and clinical studies

Although older case studies demonstrated promising results for longer gap peripheral nerve coaptation up to 15 mm with equivalent efficacy to autologous nerve transplantation, current data for that indication are sparse.^37^^,^^38^ These studies, however, have demonstrated reductions in fibrosis and neuroma formation, which have prompted continued study for this material as an adjunct nerve wrap for repair.^37^ A randomized clinical trial demonstrated significant increases in both tactile awareness through two-point discrimination and sensitivity through Semmes-Weinstein testing with the addition of chitosan nerve tubes.^39^ This study additionally reported zero neuromas formed secondary to chitosan wraps, suggesting the need for further study to elucidate if this produces clinical significance in the long run.^39^ Taken together with the promising biologic underpinnings, data support potential future use of this material as an adjunct.

## Amniotic Membrane

### Biologic basis

Human amniotic membrane has been used in a variety of medical and surgical fields and its implication in peripheral nerve repair has recently become better understood.^40^ Use of hAM is supported by its antifibrotic and proregenerative properties, as well as its source of stem cells capable of modulating neurotrophic factors.^41^ These cells provide a wide variety of regulatory mediation supporting cellular proliferation and differentiation, and inhibiting fibrosis, immune rejection, and inflammation.^41^^,^^42^ The anti-inflammatory properties of hAM additionally differentiate this material and overcome concerns of graft rejection encouraging research into its use.^41^

### Preclinical and clinical studies

These advantageous biologic properties allow hAM nerve wraps to reduce perineural fibrosis and adhesions compared with control.^14^ Animal studies have demonstrated improvements in functional recovery, histomorphometric outcomes, and reduced adhesions with use of hAM nerve wraps.^42^ One study additionally demonstrated significance in reductions in scar tissue formation, speed of functional recovery, and fiber maturation when human amniotic fluid was applied around the site of repair.^43^ Another study compared hAM with other mechanisms of coaptation and found that hAM demonstrated significantly higher numbers of axons compared with control and less fibrosis compared with both control and collagen-wrapped nerves.^41^

Clinical data for use of hAM are sparse. One study on acute traumatic injuries demonstrated improvements in regeneration and functional indices at 1 year after use of hAM as well as significant reductions in neuroma volume.^44^ A second clinical study on recurrent cubital tunnel syndrome utilized these wraps with neurolysis and demonstrated some degree of improvement in all patients with regard to pain, function, or strength without complications.^40^ These studies demonstrate that the material is safe in humans and potentially efficacious in nerve surgery; however, there remain inherent limitations within the extrapolation of these data and a significant need for further clinical investigation to understand the potential value of hAM in this space.^40^

## Conclusion

The optimal nerve coaptation adjunct remains undetermined. As summarized in Table 2, data regarding these materials largely remains heterogenous, however, each biomaterial discussed may provide various advantageous properties to enhance nerve growth around the site of coaptation. Based upon this review, current data support use of fibrin glue in conjunction with sutures, given noninferior or superior outcomes in all measurable categories in an effort to minimize potential trauma related to multiple sutures. With respect to nerve conduits, currently available evidence does not provide consistent satisfactory results with regard to patient outcomes for any specific biomaterial.^45^ Although the data reviewed demonstrate the safety of these biomaterials as well as potential mechanisms for their assistance in nerve regeneration, data are not yet sufficient to suggest for or against their use in peripheral nerve coaptation spanning end-to-end repair or during autograft or allograft reconstruction from a clinical standpoint. Additionally, the significant heterogeneity within the studies makes comparison difficult and the cumulative strength of evidence low. Further clinical trials into these materials and others must abide by standardized reporting outcomes to elucidate and compare the true value of these materials for nerve coaptation. Until then, use of these biomaterials is predicated upon a surgeon-dependent understanding of the potential risks and benefits, as described in this review, in the ongoing effort to optimize nerve repair and reconstruction.

**Table 2:** Summary of Adjunct Materials

Adjunct	Proposed Biological Basis	Summary
Fibrin glue	Catalyzes the final steps in the coagulation cascade to produce an adhesive capsule.	Produces noninferior or superior results in all outcome categories.
Type 1 collagen	Provides mechanical support for axonal outgrowth and assists in Schwann cell migration.	Heterogeneous results regarding nerve gliding, perineural scar formation, remyelination, and axonal density.
Small intestine submucosa	40% collagen; fibronectin produces adhesion; cytokines improve environment for attachment and migration with an increase promodeling macrophage type 2 response and lymphocytes; and GDNF assists with nerve regeneration.	Noninferior velocity of conduction and adhesions compared with sham control and deeper implant vascularization and fibroblast ingrowth than collagen conduits.
Chitosan	Improves survival, proliferation, and activity of Schwann cells; supports axonal outgrowth; reduces fibroblast infiltration and scarring; and produces a neuroprotective effect secondary to downstream metabolites.	Reduces fibrosis and neuroma formation and increases both tactile awareness and sensitivity to touch.
Amniotic membrane	Produces regulatory mediators that support cellular proliferation and differentiation and inhibits fibrosis, immune rejection, and inflammation.	Reduces perineural fibrosis and adhesions with improvements in functional recovery, histologic outcomes, and axonal escape.

An ideal material for nerve repair and reconstruction will be bioabsorbable while inducing a reparative inflammatory response, flexible yet resistant to collapse, and suppressive of fibrous tissue infiltration yet aiding in diffusion of neurotropic and neurotrophic factors.^46^ The coupling of meticulous microsurgical technique and this set of standards for a useful biomaterial will prompt needed advances in peripheral nerve regeneration to optimize outcomes and provide consistency of such outcomes for patients.^46^ The shortcomings of currently available materials highlight the unmet medical need and demonstrate that this area is ripe for innovation within medical devices, material science, and tissue engineering. An optimal adjunct has the potential to disrupt the field and improve outcomes for all patients.

## Conflicts of Interest

No benefits in any form have been received or will be received related directly to this article.
